# Aging Risk and Health Care Expenditure in Korea

**DOI:** 10.3390/ijerph7083235

**Published:** 2010-08-20

**Authors:** Byongho Tchoe, Sang-Ho Nam

**Affiliations:** 1 Health Insurance Review & Assessment Service, Seocho-gu, Seocho-3 dong, Seoul 137-927, Korea; 2 Korea Institute for Health and Social Affairs, Eunpyong-gu, Bulkwang-dong, Seoul 122-705, Korea; E-Mail: johnnam@kihasa.re.kr

**Keywords:** population aging, age-expenditure profile, components decomposition, panel regression, health care expenditure

## Abstract

This paper analyzes the impact of population aging on health care expenditures in Korea. Examination of the age-expenditure profile reveals that health care resources are allocated more for the older cohort of population over time, suggesting significant growth of health care expenditures due to population aging. We contend, however, that population aging is considered as a parameter rather than an independent variable to explain rising health care expenditures. This paper shows that population aging is not found to be a significant determinant of health care expenditures according to the econometric analysis using OECD health data and time-series data for Korea. Using the components decomposition method, which measures the contribution of each component of health care expenditure, we estimate that population aging contributes only less than 10 percent.

## 1. Introduction

There is a growing concern that the rapid aging of the population is a major driver of increasing health care expenditures. We can observe this from the age-expenditure profile, in which more health care monies are spent on the older cohort of the population. In order to examine the influence of population demographics on health care expenditures within particular countries, researchers have plotted health service use and/or expenditure against age, and used the resulting age-use and age-expenditure curves to quantify the relationship between age and utilization or expenditure. In OECD countries, the average *per capita* expenditures for persons aged sixty-five and older are two to eight times more than those for the working-age population and steadily increase with age [1,2].

Some studies since 1980s, however, argue that population aging is not a cause or is only a weak factor of increasing health care expenditure. The degree to which increasing expenditures are simply responses to changing demographic variables is small [3–6]. A study, utilizing age distribution of health care expenditures, showed that changes in population aging account for barely 13 percent of the total increase in health care expenditures during the period 1970–1985, and that the remainder is a result of the development of an increasing concentration of health care expenditure on older age groups [7]. Another study showed that demographic shifts and population growth accounted for only 18 percent of the observed increases in health care expenditures in England and Wales, compared to 68%, 44%, and 34% in Japan, Canada, and Australia respectively [8].

Other studies emphasize that population aging is not a major cause of increasing expenditures, and in fact, the expenditures before death are a major reason behind the high cost for old-age cohorts [3,9–14]. Predictions from a simple model that excludes time to death and uses current life tables are 9% higher than from an expanded model controlling for time to death [15]. The relationship between age, time to death and health expenditure has been extensively studied in recent years, using data from different countries. It finds no or weak age effects on health care expenditure when proximity to death is controlled for [16–18]. Age has a negligible effect on an individual’s health care expenditure both for survivors and the deceased. Conversely, proximity to death shows a strong positive relationship to an individual’s health care expenditures. Thus, the ‘red herring’ claim is vindicated by the study [19].

On the other hand, for the last several decades population aging has proceeded with economic growth in most countries. Many studies which investigate determinants of health care expenditure with comparative data of OECD countries show that some social economic factors, like income, are more significant than population aging. Cross-sectional studies of aggregate national spending levels in several countries found the percentage of the population over age sixty-five to be at best a weak predictor of expenditures [20–29].

Korea has experienced rapid aging, and the forecasts predict that Korea will soon be one of the most aged countries in the world, so population aging is often referred to as a major cause for the ever growing health care expenditures in Korea. The share of people over 65 years of age was 3.1 percent of the population in 1970, 5.1 percent in 1990, 7.2 percent in 2000 and 9.1 percent in 2005. It is predicted to reach 20.8 percent in 2028 and 38.2 percent in 2050, much higher than the 25.9 percent forecasted for other advanced countries [30]. There has been no examination of how the aging population contributes to the increase of health care expenditures in the case of Korea. This paper will attempt to fill this gap.

This study shows, first, by using age-expenditure profiles, that health care expenditure in Korea, which experiences a rapid aging, has increased with the aging population. However, this approach simply shows the relationship between age cohorts and health care expenses, and does not count other factors that influence health care expenditures other than the population aging. Thus, secondly, we investigate the determinants of health care expenditure with both cross-sectional and longitudinal data from OECD countries. Such panel analysis has been widely adopted recently. In this approach we control several factors that can influence health care expenditures, and we can then analyze separately the effects of population aging. Next, we analyze the determinants of health care expenditure in Korea using time-series data, and show the difference with the results drawn in the case of the OECD countries. Lastly, this paper measures the contribution of population aging on health care expenditures, using component decomposition method to disassemble the components that constitute the health care expenditure and then analyze how much the change in population aging affects its increase.

## 2. Methods

### 2.1. Age-Expenditure Profile Approach

The age-expenditure profile approach is limited by its need for health care expenditure data, according to age groups. Therefore, this paper uses the data produced from the National Health Insurance (NHI) in Korea. The annual health care expenditures *per capita*, by age cohort, were calculated, and thus those constituted the health care expenditure structure with age cohort. This enables us to observe how health care expenditures have changed in accordance to the age structure shifts. We measured the profiles for the years 1991, 1996, 2001, and in addition, measured a profile for 2003. Visiting days, which sum up the annual hospitalized and outpatient days by age cohort, measure the changes in service utilization by age cohort. However, when the health care expenditures by age cohort also include the price changes for services for each corresponding year, it is difficult to separate the effects of aging. Therefore, to observe the health care expenditure in accordance with the genuine change in age structure, it is necessary to find some way of standardization. The method used for this purpose is to calculate the relative indices of health care expenditure by age cohort and observe the structural changes by year. The volume of services used by patients of each age cohort can also be calculated using this method.

### 2.2. Econometric Approach Based on Regression Analysis

Researches on the factors that determine the national health care expenditures in the late 1970s and the early 1990s have usually utilized the simple or multivariable regression analysis model based on cross-sectional data. In the late 1990s, researches using various econometric models based on panel data became prevalent. The existing research results point out that the gross domestic product (GDP) *per capita* is the most influential factor that can explain changes in national health care expenditures, and in addition to income, many other socioeconomic factors and health care system related variables have been discussed in terms of how they have influenced health care costs. The influence of socioeconomic factors and health care system factors on health care expenditures has been estimated in a variety of ways, according to the methods and data used.

Table 1 summarizes the previous research results, and the income elasticity of health care expenditure appears mostly as a statistically positively significant. In addition, it has shown robust results regardless of estimation method, choice of explanatory variables, data used, or calculation of income with nominal exchange rate or purchasing power parity. However, whether the elasticity of income is greater or less than 1 has varied between research results. A population structure like aging is usually known to influence national health care expenditures insignificantly.

In this research we used the longitudinal data on 33 countries during the period between 1970 and 2001. All the countries were members of the Organization for Economic Cooperation and Development (OECD). In addition, analysis based on the time series data of Korea was compared with the analysis of OECD countries, and some implications were found. The analysis in the case of Korea has two time spans: from 1997, the year that national health insurance was introduced, until 2000, and from 1985 to 2000.

The following is the full-model used in the analysis:

(1) $${\text{NHE}}_{jt}=a_{1}+a_{2}\cdot {\text{Socio-econfactors}}_{jt}+a_{3}\cdot {\text{health}\;\text{system}\;\text{factors}}_{jt}+a_{4}\cdot \text{country}+a_{5}\cdot {\text{year}}_{j}+e_{jt}$$

In this model, NHE represents national health expenditure *per capita* and is used as a dependent variable. Independent variables are classified with socio-economic factors and health system related factors. For the socio-economic factors, we used GDP *per capita*, the ratio of people older than 65 to the total population, and the ratio of women to the whole work force. For the health system factors, we used government health expenditure, health insurance population coverage, the number of doctors per one thousand people, hospitalization cost, and new medical technology as quantitative variables. Other health system factors, such as the methods of payment to doctors and the existence of gatekeepers are included as institutional variables. These institutional variables are treated as dummy variables. In the model, j denotes the country, and t denotes the year.

We note that government health expenditure, which is mostly included in the literature in studying determinants of health care expenditure, does not include social health insurance expenditure. The term government health expenditure refers to investments in public health, which covers prevention and health improvement, prevention of pandemic diseases, public health infrastructure, and other public activities. The underlying logic of using this variable is that government investment for public health will reduce the national health expenditure.

The provider payment system shown in Table 2 needs some explanation. FFS means that doctors are reimbursed by a fee assigned for each medical service that they provided to patients. CAP means that doctors are reimbursed by the number of enrollees they care for. The insurer pays a certain amount per enrollee to doctors. WAS means that doctors are reimbursed with wage or salary paid by the insurer (usually government). Doctors are not reimbursed based on how many medical services they provided to patients, nor how many enrollees they care for.

Each quantitative variable is converted to natural logarithms to get the elasticity estimate. The logarithm transformation of variables also contributes to mitigate the heteroskedasticity of coefficients. The variables used in the models are defined in Table 2.

### 2.3. Approach to Decompose Components of Health Care Expenditure

The increase in health care expenditure can be broken down into several components: the increase in population coverage, the aging of population structure, increased fees for services, and the increased volume of services used. Through this analysis, the effect of population aging can be singled out. The data used in this analysis is the National Health Insurance data of Korea. The health insurance expenditures could be decomposed by the following equation:

(2) Health insurance expenditure=covered population×visit days per capita×expense per visit day

In Equation (2), the visit days include both outpatient visit days and inpatient days (‘length of stay’). We think the use of mixed visit days summing up both inpatient and outpatient days will simply lead to estimation errors. Separate analysis by inpatient sector and outpatient sector would be better to get more accurate estimates, because the health care utilization behavior of the elderly differs between access to hospitals (or inpatient care) and that to clinics (or outpatient) by country. We needed, however, to simplify the model, and considered that elderly patients with chronic diseases will stay longer in bed and visit doctors more for outpatient care. Thus we believe that the variable ‘visit days’ used in the model, which aggregates inpatient days and outpatient visit days, will represent the increasing volume of utilization according to population aging.

What we want to know in Equation (2) is how much the health insurance expenditures are affected by the population aging, which can be expressed as “demographic structure change.” The visit days per *capita* are likely to be extended longer as people are getting older. The expense per visit day is also supposed to increase according to the population aging. Both the extension of visit days and the increase of expense, however, are not only caused by the population aging. Thus both of them are decomposed of the factor affected from the demographic structure change and the remaining part, such as Equations (3) and (4).

(3) Visit days per capita=demographic structure change [1]×adjusted visit days per capita

(4) Expense per visit day=demographic structure change [2]×adjusted expense per visit day

In Equations (3) and (4), we need some description of the demographic structure change [1,2]. The demographic structure change [1] means the effect of demographic structure change (‘population aging’) on the visit days *per capita*. In Equation (3), the visit days *per capita* are composed of the effect of demographic structure change on the visit days, which is called “demographic structure change [1]”, and the remaining part which is “adjusted visit days *per capita*” in Equation (3). The demographic structure change [2] means the effect of demographic structure change (‘population aging’) on the expense per visit day. In Equation (4), the expense per visit day is composed of the effect of demographic structure change on the expense, which is called “demographic structure change [2]”, and the remaining part which is “adjusted expense per visit day” in Equation (4).

The adjusted expense per visit day is composed of the conversion factor (‘fee’), which converts the score of each service item based on the resource based relative value scale (RBRVS) into price of charge, and the remaining part, such as the Equation (5).

(5) Adjusted expense per visit day=fee×readjusted expense per visit day

We note here that “fee” in Equation (5) includes only service charges, which exclude cost of drugs and supplies, but include doctor’s service fees and expenses for operating the institution. Some drugs and supplies are included in medical service charges, though. The share of service charges is around 0.68 taken from the whole expenses. Therefore, Equation (2) may be written again like Equation (6):

(6) Health insurance expenditure=covered population×(demographic structure change [1]×adjusted visit days per capita)×(demographic structure change [2]×fee×readjusted expense per visit day)

Equation (6) can be transposed into Equation (7) with natural logarithm.

(7) Rate of change in health insurance expenditure = rate of change in covered population + (rate of change in demographic structure change [1] + rate of change in adjusted visit days per capita) + (rate of change in demographic structure change [2] + rate of change in fee + rate of  change in readjusted expense per visit day)

We should notice here that “rate of change in fee” and “rate of change in readjusted expense per visit day” include price inflation. Therefore, those rates of change in fee and expense can be expressed in real terms separated from price inflation. The rates of change in demographic structure can be expressed as changes from time point *a* to time point *b*.

The data for the calculation is provided in Table 3. The data are the number of population by age group, annual visit days per person by age group, and expense per day per person by age group for the three years, 1991, 1999 and 2003. These numbers show how the demographic structure changes into population aging, and show how the volume of health care utilization and their expenses change in response to the population aging.

The rates of change in demographic structure change from the Equation (7) can be expressed like Equations (8) and (9), where *j* denotes age cohort.

(8) $$\text{Rate}\;\text{of}\;\text{change}\;\text{in}\;\text{demographic}\;\text{structure}\;\text{change}\;[1]=[\sum_{j}{\text{covered}\;\text{population}}_{b,j}\times ({\text{covered}\;\text{population}}_{a}/{\text{covered}\;\text{population}}_{b})\times {\text{visit}\;\text{days}\;\text{per}\;\text{capita}}_{a,j}]/[\sum_{j}{\text{covered}\;\text{population}}_{a,j}\times {\text{visit}\;\text{days}\;\text{per}\;\text{capita}}_{a,j}]$$

(9) $$\text{Rate}\;\text{of}\;\text{change}\;\text{in}\;\text{demographic}\;\text{structure}\;\text{change}\;[2]=[\sum_{j}{\text{visit}\;\text{days}}_{b,j}\times ({\text{total}\;\text{visit}\;\text{days}}_{a}/{\text{total}\;\text{visit}\;\text{days}}_{b})\times {\text{expense}\;\text{per}\;\text{visit}\;\text{day}}_{a,j}]/[\sum_{j}{\text{visit}\;\text{days}}_{a,j}\times {\text{expense}\;\text{per}\;\text{visit}\;\text{day}}_{a,j}]$$

Rate of changes in adjusted visit days *per capita* in Equation (7) can be expressed in Equation (10), where change in visit days is separated from change in demographic structure.

(10) Rate of change in adjusted visit days per capita=rate of change in visit days per capita÷rate of change in demographic structure change [1]

And rate of change in readjusted expense per visit day can be expressed in two steps. In the first step, change in adjusted expense is separated from the change in demographic structure, as in Equation (11). In the second step, change in readjusted expense is separated from change in service fees, as in Equation (12).

(11) Rate of change in adjusted expense per visit day = rate of change in expense per visit day÷rate of change in demographic structure change [2]

(12) Rate of change in readjusted expense per visit day=rate of change in adjusted expense per visit day÷rate of change in fee level

Three periods of analysis were selected: 1991 to 1999, 1999 to 2003, and 1991 to 2003. The reason we divided the data into three periods is that the separation of prescribing and dispensing drugs was introduced in July 2000, and that policy had a striking impact on the national health care expenditures.

## 3. Results

### 3.1. Age-Expenditure Profile Analysis

**Figure 1.**
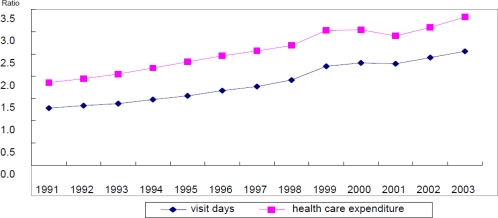
Ratio of health care utilization of 65+ person to 65– person, in terms of health care expenditures and visit days per capita.

As the proportion of older population increases, their health care expenditures tend to increase accordingly, where the health care expenditure represents those accrued in the national health insurance that includes doctors’ service charges and operating expenses, but excludes expenditures for drugs and supplies. The ratio of the health care expenditure of person over 65 to that of person under 65 during the period between 1991 and 2003 is shown in Figure 1.

The cause of this phenomenon is that persons over 65 have more health care needs than those under 65. However, the costs of fulfillment of the health care needs of those over 65 can be met as a burden on the population labor force. Thus, as the productivity of the labor force improves and income levels get higher, it becomes easier to fulfill the health care needs of older persons.

**Figure 2.**
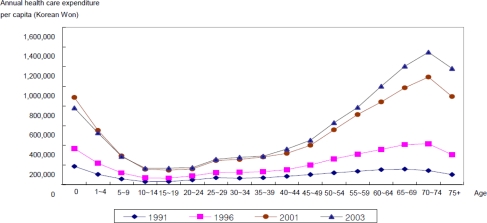
Structure of age-health care expenditure profile (current prices).

When we look at the trend of health care expenditure by age groups, we see that the health care expenditure *per capita* is high for infants and children; it decreases among the youth, it increases in middle age, and then it increases rapidly in later years. On a graph, this trend is a U-shaped curve, as shown in Figure 2. When measured in a five year gap, the U curve develops a steep valley.

**Figure 3.**
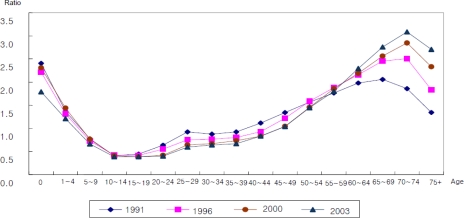
Structure of age-health care expenditure profile (standardization). Note: The unit of value of vertical axis is a ratio of health care expenditure at each age group to the weighted average of health care expenditures for all age groups, where the weighted average is standardized as 1.0.

To correct the problem that occurs when comparing with current prices of each year, the relative magnitude of health care expenditures by ages in each year is calculated, as shown in Figure 3. Compared to the health care expenditures of 1991, those of 2003 were relatively higher for older persons and relatively lower for younger persons. Especially for youth and middle-aged people, health care expenditures were relatively low. This shows that as the data moves closer to the present, the health care expenditures for older patients are increasing. This implies also that health care resources distributed for older people are increasing. So to extend the lives of those who are older, the structure of health care resources allocation is undergoing changes.

A question that arises is whether the volume of health care services used is following a trend similar to that of health care expenditures. The expense per service unit applied to older people could be increased.

**Figure 4.**
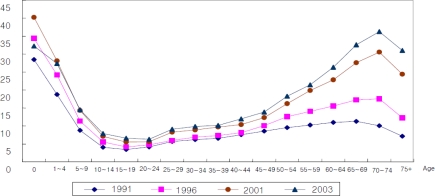
Structure of age-visit days profile.

Even without these price-related factors, we can observe whether the steep U shaped form could stay the same. As shown in Figure 4, as population aging progresses in the whole age structure, the visit days increase, and the number of visit days for older people increases faster.

**Figure 5.**
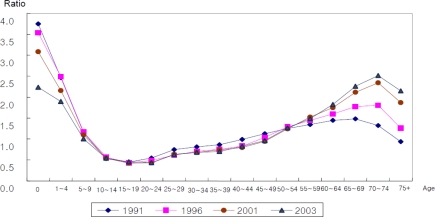
Structure of age-visit days profile (standardization). Note: The unit of value of vertical axis is a ratio of visit days at each age group to the weighted average of visit days for all age groups, where the weighted average is standardized as 1.0.

The visit days, with the average growth of visit days subtracted, are shown in Figure 5. The visit days increase among older people and decrease relatively among the young, showing a similar pattern as the changes in health care expenditure. Thus, even on the quantitative structure without the price factors, the older cohort consumes a greater share of health care resources.

From these observations, is it possible for us to regard population aging as a decisive factor which results in the increase of health care expenditures? The answer is not that simple. So far we have only observed the relationship between population aging and health care expenditures. For instance, as population aging continues, we see health care expenditures increase for people who are 70 years old. However, the causes of the increased expenditures are complicated; as the national income rises, there could be more resources available involved in curing older people, and advances in medical technology could have invested more in health care for them. Since the national income grows accompanied with technological advancement from the perspective of economic history, the phenomenon of rising health care expenditures may not be explained by population aging *per se*, but rather by the increase in available national income that implies more resources for health care. In addition, if the extended life span is closely related with the rising national income, the rise of health care expenditures toward older cohorts will be related to the growing national income. Strictly speaking, population aging may not be an independent variable that influences health care expenditures. Population aging progresses in response to improvement in health care, better nutrition, higher education, health awareness, and so on. Therefore, the rise in health care expenditures could be explained by the causes that make population aging possible, rather than by population aging *per se*.

### 3.2. Panel Analysis Determining National Health Expenditures

The panel analysis for the OECD countries shows that the results are influenced by the period of analysis chosen and the choice of explanatory variables used. Population aging does not appear to be a significant variable in determining national health expenditures. However, in Model 2, shown in Table 4, population aging seems to be significant under the significance level of 10 percent, but the elasticity is only 0.16, which is not large in magnitude. The elasticity means, here, the percentage change in the national health expenditure (dependent variable) in response to a one percent change in the relevant independent variable holding constant all the other determinants of national health expenditures. The determining factors that significantly affect the national health care expenditure are both *per capita* income and women’s participation in the labor force, although the elasticity varies widely because the model is constructed differently.

It is curious that the signs of variables “doctor” and “inpatient” are inconsistent in Models 2 and 3 in Table 4. The difference between the two models is whether or not the variables such as payment methods and gatekeeper are included in the model. We believe Model 3 stands to reason, so we can interpret that an increased supply of doctors leads to higher health care expenditures when health care systems like payment methods and gatekeeper system are taken into account in the model. It is sensible that the higher inpatient expenditure leads to more health care expenditure, but the result appears to be opposite in Model 3, even though the size of the coefficient is very small. We need more investigation of how the independent variables including “doctor” and “inpatient” influence each other and affect national health care expenditures with the existence of different health care systems in each country.

The results from the time-series data in Korea are shown in Table 5. When we regard the Model 1 as trustworthy because it covers a long period, the result shows that population aging is an insignificant factor. The variables such as income, health insurance coverage, and number of doctors were statistically significant in 5 percent significance level. The income elasticity, especially, was close to 1. The elasticity of insurance coverage expansion was positive (+0.228), which is inconsistent with the results from the other OECD countries. This is because Korea expanded the insurance coverage of the population in a short period, leading to a sharp increase in health care expenditure.

It is curious that the increase in the number of doctors has an effect on containing health care expenditures. This result is opposite to that seen in other OECD countries. Korea rejects the supplier-induced demand hypothesis which claims that the increase of doctors is the main cause of the rise of health care expenditures. Therefore, we can conclude temporarily that the consumer’s expanded demand, which was originated by the expansion of the insurance-covered population, is a stronger factor than the supplier-induced demand for health care. However, women’s participation in the work force did not affect health care expenditures significantly. In Model 2 conducted for the recent period, income and health insurance coverage of the population were both significant, but population aging was not.

In conclusion, the leading cause of rising health care expenditure is related to the increase in national income and the expansion of the health insurance covered population. The increase in the number of doctors was influential in containing the increase in health care expenditure, and population aging was not significant, as in other OECD countries.

### 3.3. Components Decomposition of Health Care Expenditure

When the period from 1991 to 2003 is selected, the contribution of population aging to the rise in health care expenditure appears to be 6.6 percent, as shown in Table 6, which presents the result that includes the price inflation as a factor contributing to the rise of health care expenditure. This table shows that the contribution of change in demographic structure was 6.6 percent in 1991 to 2003, and 9.51 percent in 1999 to 2003, which shows that population aging became a more influential factor in medical expenditure increases. The effect of population aging on the rise of health care expenditure is less than 10 percent. The volume of health care utilization, which is a quantitative factor, affects the health care expenditure increase by around 33 percent. The increased volume reflects both the increased demand of health care services according to higher income level and the induced demand from the suppliers, and also includes the expansion of health care use originated by the longer period of insurance coverage. The contribution of increased fee levels is around 39 percent, but after the introduction of the separation policy of prescribing and dispensing drugs, increased fee levels contributed up to around 47 percent. The contribution of cost per visit day after adjusting the increasing fees is around 13.8 percent, but after the introduction of the separation policy the influence decreased drastically. The expense per visit day includes the increasing cost of pharmaceuticals and medical supplies and will also include the intensity of treatment and the services increased due to the expanded coverage of health insurance. However, as the fee and the expense per visit day include increased price, their contribution to rising health care expenditure includes a natural increase by the price index.

Table 7 separates the price inflation from the health care fees component, and analyzes the breakdown of health care expenditures. The contribution of changes in demographic structure is almost identical. On the one hand, the contribution of raised price inflation was about 28.4 percent in 1991 through 2003, and the contribution of medical fees in terms of real price was about 24.4 percent. On the other hand, the contribution of expense per visit day was almost zero. Especially, after the separation policy, the contribution of fees was very large, and the contribution of expense per visit day resulted in a negative (−) value. This shows that the policy containing health care expenditure was strong enough to escape from the financial crisis resulting from the separation policy.

The results after excluding the price inflation are shown in Table 8. All terms are converted into real prices by subtracting price inflation. The contribution of change in demographic structure is about 9.2 percent for the period 1991–2003, but for the recent period 1999–2003 it is 11.4 percent, illustrating further growth in population aging. The largest contributor is health care utilization volume, and its contribution is about 46.4 percent for the period 1991–2003. This is because, first, the demand for health care increased as the income levels rose; second, after the introduction of health insurance, the burden of individual payment decreased and the demands for health care increased; and third, the insurance coverage widened. The contribution of medical fees level, which is about 34.1 percent, is impressive. This contribution was only about 14.9 percent for the period 1991–1999 before the introduction of separation of prescribing and dispensing drugs. It is clear that increased fees led the fast growth of health care expenditure after the introduction of the separation policy.

Population aging contributed less than 10 percent to the increase of health care expenditures. Health care expenditures increased mainly due to the increase in both the volume of utilization and the increasing fees. This implies that the higher level of income and the introduction and/or expansion of health insurance affected the increase in health care utilization, and in turn the increased utilization produced the increase in health care expenditure.

## 4. Discussion and Conclusions

The objective of this paper was to analyze the impact of population aging on health care expenditures in the case of Korea. We applied three approaches to do this. The first approach using age-health care expenditure profiles showed that, when we look at the profiles closer to the present, the health care expenditures for the older age groups came to increase further. The health care utilization excluding price index, like GDP deflator, was also increasing more rapidly for the older age cohorts. This implies that the health care resources that are allocated to the older age groups are growing, due to the increased resources applied to extend the expected life span that could be generally enabled through the higher national income level, rather than the population aging *per se*. Therefore, population aging may not be an independent variable that contributes to the rise in health care expenditure. Instead, the factors that make population aging possible such as income, education, and sanitation will be the ultimate determining factors.

Some research attributes most of the rise in health care expenditure to the population aging, explaining it by citing the Sisyphus syndrome. This research claims that health care expenditures increase leads to a longer life span (‘provokes aging’) and that the extended life span leads to a higher demand for public health services and finally increases the health care expenditure [31]. The former relates to the discussion of health production function that explains that the increase in health care expenditure will increase the expected life span, and the latter is explained by the relationship that as older people continue to age, their political influence increases. However, a regression analysis using OECD health data was not able to prove that hypothesis [32]. Later, another study tried the regression analysis again with the same data source [33]. During the period of 1970 through 1991, the Sisyphus syndrome existed, but in 1992 through 1999 it disappeared. In the case of Korea, a study verified that the Sisyphus syndrome exists for the period from 1977 to 1998, but in later years, negative effects became influential and the results showed no Sisyphus syndrome [34]. Therefore, it was difficult to verify the Sisyphus syndrome statistically because historically population aging coincided with economic growth, and the increased health care expenditures have been explained mostly by the increased rate of income. In the case of Korean study, we have some reservations about accepting the result. Older people generally do not have enough power to develop political influence in Korea, and considering that income level rose faster than the speed of population aging, the conclusion that population aging is a factor in increasing health care expenditure seems unusual.

To overcome the limitation of neglecting other factors besides population aging in observing the health care expenditure profile by age, we applied the second approach, in which we used longitudinal regression analysis to OECD countries. The result of this analysis shows that in OECD countries, generally population aging was not a significant variable contributing to increased health care expenditures. A similar result was also shown in the case of Korea, though a relatively short period of time-series data was used. The analyses of relatively recent periods, which show a somewhat faster aging, show also that population aging was not a significant factor in the increase of health care expenditures. The main causes of increased expenditures are thought to be both the national income and the expanded application of national health insurance. In the approaches using regression analysis, the effect of higher income on health care expenditure increase could have overwhelmed the influence of population aging in determining health care expenditure. In this approach, we cannot verify, however, of how much the population aging contributes to the increase of health care expenditure.

We used an alternative approach, in which we broke down the components contributing to increased health care expenditure and singled out the contribution of the change in demographic structure, most of which is accrued from population aging. During the period of 1991 to 2003, the population aging contributed only 6.6 percent to increasing health care expenditures. However, in real terms excluding price inflation such as GDP deflators, the contribution of population aging was 9.2 percent. In this approach, the contribution of population aging to increased health care expenditures was considered less than 10 percent. The main drivers of health care expenditure increases appeared to be both the increased utilization of volume and the increased fee levels. The real drivers beneath these factors were thought as the higher national income and the introduction of national health insurance. Several studies show that the effects of aging population may be overwhelmed by other factors like the introduction of new technologies and treatments [35–37]; increased utilization, for example, of drugs and diagnostic tests; and price inflation [38,39]. It can also be considered that at the aggregate level, national income is closely related to the use of new technology and product. The relationship between health expenditure and national income is supposed to be important from the broader perspective.

In short, the influence of population aging on the rise in health care expenditures can be viewed as superficial. The reality is that the factors that cause population aging contribute to higher health care expenditure. A study suggested that the fixation on population aging provides an ‘illusion of necessity’ [40]. By making it seem as though health care expenditure is inevitable in higher age, attention is diverted from the real causes of growth of the health sector. These are technical progress in medicine, the secular increase in income, and wrong incentives for providers and consumers of health care caused by government regulation and extensive social health insurance coverage [40]. In other words, blaming the population aging serves as a ‘red herring’, distracting from choices that ought to be made to curb the steadily rising health care expenditures in the Western world [41]. Another study emphasizes that health care expenditure might be driven more by changes in the propensity to move into long-term care and medical technology than age and gender alone as often claimed in public discussion [42]. Thus the future expenditure is more likely to be determined by health policy actions than inevitable trends in the demographic composition of the population. Reduced mortality and low growth in the expenditure, when cost of dying is excluded, could reduce forecasted expenditures, but high growth in expenditures for those not close to death and for non-hospital services could create new economic pressures on health care systems [43]. Though health care expenditure is associated with proximity to death, the effect of income could be more important among individuals who were close to death.

In conclusion, population aging is thought to be an endogenous variable rather than an independent variable in how it impacts health care expenditures. It seems to be a parameter or mediator in the process of explaining the causes of increasing health care expenditures. This paper, of course, has some limitation. Time span for the estimation of the determinants of national health expenditure in Korea is not sufficiently long. We need more data for the longer period extending up to date. But when we extend the period, we will have a difficulty to manage in the model, the financial shock that happened in 2001 due to the sudden increase of medical fees that was caused by the sudden introduction in July 2000 of the policy of separating prescribing and dispensing. In the analysis of components decomposition, we took the components which contribute to the increase of health care expenditure as population aging, price, volume, and intensity. But the components can be separated in more detail. Especially the effect of coverage expansion of benefits and the effect of increasing patient (or prevalence rate) can be separated from the above components. It is, however, technically difficult to separate them in an acceptable manner. How the other important factors, such as cost of dying, diffusion of high technologies, growth of health care provision market are related with population aging will be promising issues of further study in Korea.

## Figures and Tables

**Table 1.** Results from the studies of the determinants of national health expenditures.

	Newhouse [27]	Leu [26]	Gerdtham [21]	Gerdtham [22]	Gerdtham [23]	Hitiris and Posnett [25]	Gerdtham [24]	Barros [20]	Roberts [28]

Data	13 countries 1971 cross-sectional	19 countries 1974 cross-sectional	19 countries 1987 cross-sectional	19 country panel (1974, 80, 87)	22 country panel (1972∼87)	20 country panel (1960∼87)	22 country panel (1970∼91)	24 country panel (1960∼90)	20 country panel (1960∼93)
Per capita GDP	Above 1.0	1.1∼1.3	1.33	1.27	0.74	1.026 (exchange rate) 1.16 (PPP)	0.74	below 1.0	above 1.0
Share of public finance	-	0.2∼0.3	–0.48	-	–0.12	-	Not significant	Not significant	0.7
Share of 65+ population	-	-	-	-	-	-	Not significant	Not significant	Not significant
Price index	-	-	-	-	–0.16	-	-	-	-
Share of inpatients exp.	-	-	0.22	0.31	-	-	0.06	-	-
No. of doctors	-	-	-	-	-	-	–0.14^1^	-	-
Share of public beds	-	0.8∼0.9	-	-	-	-	–0.32	-	-
NHS	-	–0.2∼–0.24	-	-	-	-	-	-	-
Primary care	-	-	-	-	-	-	–0.18	Not significant	-
Fee-for-service	-	-	1.12	1.13	-	-	Not significant	-	-

Note 1: not significant under the fee-for-service payment model.

**Table 2.** Definition of variables on the determinants of national health expenditures.

	**Variables**	**Definition**

Quantitative variables	NHE	*Per capita* real national health expenditures (PPP 1995 US dollars)
	GDP	*Per capita* real GDP (PPP US dollar of 1995)
	GOV	*Per capita* real government health expenditure (PPP 1995US dollars)
	coverage	National health insurance covered population of total population (%)
	doctor	Number of doctors per one thousand people
	inpatient	Share of inpatient expenditures of national health expenditures (%)
	65 over	Share of 65+ population of total population (%)
	flabor	Share of female labor forces (%)
	TEC	New technologies (number of CT, MRI)

Provider payment system	FFS	Fee-for-service (dummy variable)
	CAP	Capitation (dummy variable)
	WAS	Wages and salaries (dummy variable)

Primary care	GTK	Existence of gatekeepers (dummy variable)

**Table 3.** Data for calculation of demographic structure change by age cohorts.

	**1991**			**1999**			**2003**		

Age group	Population	Visit days *per capita* (Annual)	Expense per day (Korean Won)	Population	Visit days *per capita* (Annual)	Expense per day (Korean Won)	Population	Visit days *per capita* (Annual)	Expense per day (Korean Won)

0	596,197	28.49	6,584	542,215	37.96	13,568	440,422	32.23	24,208
1∼ 4	2,440,863	18.76	5,685	2,643,523	27.31	11,643	2,270,202	27.37	19,262
5∼9	3,333,088	8.77	6,900	3,389,172	13.19	13,269	3,335,953	14.51	19,942
10∼14	3,676,286	4.04	7,864	2,987,566	6.07	15,513	3,285,293	7.86	21,406
15∼19	3,962,564	3.42	10,135	3,675,729	4.73	20,344	3,023,589	6.58	25,611
20∼24	4,118,905	4.15	11,966	3,672,719	5.12	22,927	4,019,538	6.29	27,734
25∼29	4,128,382	5.68	12,661	4,514,821	6.81	25,100	3,913,403	9.09	28,555
30∼34	4,220,936	6.19	11,048	4,309,290	7.64	22,849	4,570,971	9.85	28,379
35∼39	3,201,607	6.56	10,967	4,270,569	8.41	22,525	4,260,392	10.14	28,736
40∼44	2,317,100	7.54	11,526	3,776,972	9.13	23,605	4,329,836	11.94	30,482
45∼49	2,037,280	8.56	12,210	2,638,961	11.21	24,855	3,590,153	13.88	32,591
50∼54	1,947,689	9.52	12,829	2,172,347	14.12	26,184	2,511,498	18.26	34,547
55∼59	1,570,815	10.23	13,455	2,030,856	17.79	27,451	2,054,699	21.45	36,626
60∼64	1,126,154	10.99	14,052	1,699,636	20.53	28,376	1,955,699	26.35	37,993
65∼69	837,432	11.26	14,265	1,154,766	24.15	28,182	1,482,244	32.61	36,923
70∼74	584,943	10.05	14,423	768,481	26.85	27,653	944,519	36.26	37,131
75∼79	644,908	7.13	14,706	936,328	21.52	28,622	1,114,375	30.99	38,107

Sum or average	40,745,149	7.60	10,254	45,183,951	11.64	21,642	47,102,786	14.43	30,211

Note: The exchange rate of Korean currency is around 1,200 Won/US dollar.

**Table 4.** Determinants of national health expenditures in OECD countries.

**Variable**	**Model 1(1984–2001)**	**Model 2(1970–2001)**	**Model 3 (1970–2001)**	**Pooled Regression Results (1984–2001)**

ln(GDP)	0.588**	0.814**	1.043**	1.147**
ln(GOV)	0.261**	0.203**	−0.004	−0.007
ln(coverage)	0.043	−0.002	−0.275**	−0.320**
ln(doctor)	−0.104	−0.303**	0.534**	0.224**
ln(inpatient)	0.082**	0.144**	−0.094*	0.153*
ln(65over)	0.140	0.160*	−0.020	0.089
ln(flabor)	1.261**	0.274**	0.565**	0.612**
ln(TEC)	0.075**	-	-	−0.027
FFS	-	-	0.185**	-
CAP	-	-	0.106**	-
WAS	-	-	0.086**	-
GTK	-	-	0.049**	-

No. of observation (No. of countries)	120 (33)	283 (33)	283 (33)	120 (33)
R^2^	0.995	0.992	0.956	0.939
Degree of freedom	111	225	240	111

Characteristics	Two-way Random Effect Model	Two-way Fixed Effect Model	Two-way Fixed Effect Model	-

Note:

** p < 0.05,

* p < 0.1.

**Table 5.** Determinants of national health expenditures in Korea.

	**Model 1 (1977–2000)**	**Model 2 (1985–2000)**	**Model 3 (1985–2000)**

ln(GDP)	0.962**	1.041**	0.958**
ln(GOV)	-	-	0.287*
ln(coverage)	0.228**	0.300**	0.148
ln(doctor)	−0.996**	−0.886	−1.117**
ln(inpatient)	-	-	−0.288
ln(65over)	0.304	1.691	0.799
ln(flabor)	−0.321	−1.169	−1.621
Year	0.056*	0.001	0.036

R^2^	0.988	0.983	0.990
F-value	229.769	84.732	85.198
No. of observation (degree of freedom)	24 (17)	16 (9)	16 (7)
D-W statistics	1.953	2.404	2.690

Note:

** p < 0.05,

* p < 0.1.

**Table 6.** Decomposition of increasing health care expenditures by components, including price inflation in the medical fees (in percent).

**Period**	**Population**	**Demographic change (aging)**	**Adjusted visit days per capita**	**Medical fees level**	**Readjusted expense per day**	**Sum**

1991–1999	7.98	6.00	31.29	32.11	22.61	100
1999–2003	6.90	9.51	35.23	46.89	1.47	100
1991–2003	7.64	6.60	33.15	38.84	13.77	100

**Table 7.** Decomposition of increasing health care expenditures by components, separating price inflation with the medical fees (in percent).

**Period**	**Population**	**Demographic change (aging)**	**Adjusted visit days per capita**	**Price (GDP deflator)**	**Medical fees level**	**Readjusted expense per day**	**Sum**

1991–1999	8.00	6.01	31.33	34.18	9.82	10.66	100
1999–2003	6.93	9.55	35.35	16.24	37.54	−5.61	100
1991–2003	7.66	6.61	33.23	28.44	24.41	−0.35	100

**Table 8.** Decomposition of increasing health care expenditures by components, excluding price inflation from the medical fees (in percent).

**Period**	**Population**	**Demographic change (aging)**	**Adjusted visit days per capita**	**Fees**	**Readjusted expense per day**	**Sum**

1991–1999	12.15	9.13	47.60	14.92	16.19	100
1999–2003	8.27	11.40	42.21	44.82	−6.69	100
1991–2003	10.70	9.24	46.43	34.12	−0.49	100

## References

[1] CMS (Centers for Medicare and Medicaid Services) Personal Health Care Expenditures by Age: 1987–1999 U.S. Department of Health and Human Services Washington, DC, USA 2006

[2] Mayhew L Health and Elderly Care Expenditure in an Aging World International Institute for Applied Systems Analysis Laxenburg, Austria 2000

[3] Fuchs VR ‘Though much is taken’: reflections on aging, health and medical care Milbank Mem. Fund Quart.-Heal. S 1984 62 142 166 6425716

[4] Hertzman C Hayes M Will the elderly really bankrupt us with increased health care costs? Can. J. Pub. Health 1985 76 373 377 4092178

[5] Barer ML Evans RG Hertzman C Aging and health care utilization: New evidence on old fallacies Soc. Sci. Med 1987 24 851 862 3616679 10.1016/0277-9536(87)90186-9

[6] Getzen TE Population aging and the growth of health expenditures Journal of Gerontology 1992 47 s98 104 1573213 10.1093/geronj/47.3.s98

[7] Gertham UG The impact of aging on health care expenditure in Sweden Health Policy 1993 24 1 8 10125808 10.1016/0168-8510(93)90083-2

[8] Seshamani M Gray A The impact of ageing on expenditures in the National Health Service Age Ageing 2002 31 287 294 12147567 10.1093/ageing/31.4.287

[9] Bloom BS Kissick PH Home and hospital costs of terminal illness Med. Care 1980 18 560 564 7401709 10.1097/00005650-198005000-00009

[10] Scitovsky AA ‘The high cost of dying’ revisited Milbank Quart 1994 72 561 591 7997219

[11] Lubitz J Prihoda R The use and costs of medicare services in the last 2 years of life Health Care Finan. Rev 1984 5 117 131 PMC4191360 10310597

[12] Lubitz J Riley GF Trends in Medicare payments in the last year of life N. Engl. J. Med 1993 328 1092 1096 8455667 10.1056/NEJM199304153281506

[13] Mendelson DN Schwartz WB Effects of aging and population growth on health care costs Health Affair 1993 12 119 125 10.1377/hlthaff.12.1.119 8509013

[14] Kimberlyn M Green B Barer ML Evans RG Hertzman C Normand C Age, costs of acute and long-term care and proximity to death: Evidence from 1987–88 and 1994–95 in British Columbia Age Ageing 2000 29 249 253 10855908 10.1093/ageing/29.3.249

[15] Stearns SC Norton EC Time to include time to death? The future of health care expenditure predictions Health Econ 2004 13 315 327 15067670 10.1002/hec.831

[16] Seshamani M Gray AM Aging and health care expenditure: The red herring argument revisited Health Econ 2004 13 303 314 15067669 10.1002/hec.826

[17] Seshamani M Gray AM A longitudinal study of the effects of age and time to death on hospital costs J. Health Econ 2004 23 217 235 15019753 10.1016/j.jhealeco.2003.08.004

[18] Werblow A Felder S Zweifel P Population Aging and Health Care Expenditure: A School of ‘Red Herring’? Working Paper No. 11; Faculty of Economics and Management Magdeburg Magdeburg, Germany 2005

[19] Werblow A Felder S Zweifel P Population ageing and health care expenditure: A school of ‘red herrings’? Health Econ 2007 16 1109 1126 17311357 10.1002/hec.1213

[20] Barros PP The black-box of health care expenditure growth determinants Health Econ 1998 7 533 544 9809710 10.1002/(sici)1099-1050(199809)7:6<533::aid-hec374>3.0.co;2-b

[21] Gerdtham UG Sogaard J Anderson F Jonsson B Econometric analysis of health expenditure: A cross-sectional study of the OECD countries J. Health Econ 1992 11 63 84 10119757 10.1016/0167-6296(92)90025-v

[22] Gerdtham UG Sogaard J Jonsson B Anderson F A pooled cross-section analysis of the health care expenditure of the OECD countries Health Economics Worldwide Zweifel P Frech HE III Kluwer Academic Publishers Dordrecht, The Netherlands 1992 287 310 10.1007/978-94-011-2392-1_14 10172496

[23] Gerdtham UG Jonsson B International comparisons of health care expenditure: Conversion factor instability, heteroscedasticity, outliers and robust estimators J. Health Econ 1992 11 189 197

[24] Gerdtham UG Jonsson B MacFarlan M Oxley H The determinants of health expenditure of the OECD countries Health, the Medical Profession, and Regulation Zweifel P Kluwer Academic Publishers Dordrecht, The Netherlands 1998 113 134

[25] Hitiris T Posnett J The determinants of effects of health expenditure in developed countries J. Health Econ 1992 11 173 181 10122977 10.1016/0167-6296(92)90033-w

[26] Leu RE The public-private mix and international health care cost Public and Private Healthcare Services Culyer AJ Jonsson B Basil Blackwell Oxford, UK 1986 41 63

[27] Newhouse JP Medical care expenditure: A cross-national survey J. Hum. Resour 1977 12 115 125 404354

[28] Roberts J Sensitivity of elasticity estimates for OECD health care spending: Analysis of a dynamic heterogeneous data field Proceedings of the Seventh European Workshop of Econometrics and Health Economics Helsinki, Finland 9–12 September 1998 10.1002/(sici)1099-1050(199908)8:5<459::aid-hec454>3.0.co;2-u 10470551

[29] Reinhardt UE Hussey PS Anderson GF Cross-National comparisons of health systems using OECD data Health Affair 2002 21 169 181 10.1377/hlthaff.21.3.169 12025981

[30] Bureau of Statistics Long-term Population Projection in Korea Government of the Repulic of Korea Daejon, Korea 11 2006 (in Korean).

[31] Zweifel P Matteo F Is there a Sisyphus Syndrome in Health Care? Health Economics Worldwide Zweifel P Frech HE III Department of Health Economics and Public Policy Series; Kluwer Academic Publishers Dordrecht, The Netherlands 1992 1 311 330 10.1007/978-94-011-2392-1_15 10172497

[32] Zweifel P Lukas S The Sisyphus Syndrome in Health Revisited Socioeconomic Institute, University of Zurich Zurich, Switzerland 2002

[33] Ryu KC Kim EJ Lim JY Sisyphus syndrome in health care cost Korean J Health Econ Policy 2005 11 63 96 (in Korean).

[34] Tchoe B Nam SH Shin YJ The determinants of national health expenditures Korean Rev Health Policy Admin 2004 14 99 116 (in Korean).

[35] McClellan M Are the returns to technological change in health care declining? PNAS 1996 93 12701 12708 8917482 10.1073/pnas.93.23.12701 PMC34125

[36] Cutler DM McClellan M Is technological change in medicine worth it? Health Affair 2001 20 11 29 10.1377/hlthaff.20.5.11 11558696

[37] Meara E White C Cutler DM Trends in medical spending by age, 1963–2000 Health Affair 2004 23 176 183 10.1377/hlthaff.23.4.176 15318578

[38] Goetghebeur MM Forrest S Hay JW Understanding the underlying drivers of inpatient cost growth: A literature review Am J Manag Care 2003 9 SP3 12 12817611

[39] Koenig L Siegel JM Dobson A Hearle K Ho S Rudowitz R Drivers of healthcare expenditures associated with physician services Am J Manag Care 2003 9 SP34 42 12817614

[40] Evans RG Illusion of necessity: Evading responsibility for choice in health care J. Health Polit. Policy Law 1985 10 439 467 3935713 10.1215/03616878-10-3-439

[41] Zweifel P Felder S Meier M Ageing of population and health care expenditure: A red herring? Health Economics 1999 8 485 496 10544314 10.1002/(sici)1099-1050(199909)8:6<485::aid-hec461>3.0.co;2-4

[42] Hakkinen U Martikainen P Noro A Nihtila E Peltola M Aging, Health Expenditure, Proximity of Death and Income in Finland Discussion Papers 1/2007; STAKES Helsinki, Finland 2007 10.1017/S174413310800443X 18634626

[43] Payne G Laporte A Deber R Coyte PC Counting backward to health care’s future: Using time-to-death modeling to identify changes in end-of-life morbidity and the impact of aging on health care expenditures Milbank Quart 2007 85 213 257 10.1111/j.1468-0009.2007.00485.x PMC2690327 17517114

